# Diurnal temperature range: A climatological primer for health researchers

**DOI:** 10.1371/journal.pone.0352866

**Published:** 2026-07-07

**Authors:** Robert Davis, Grace Delaar, Kaleb Notari, Mia McCarrick, Parker Sims, Jane Ruggles, Julia Cardwell, Charles Konrad, Chris Fuhrmann

**Affiliations:** 1 Department of Environmental Sciences, University of Virginia, Charlottesville, Virginia, United States of America; 2 Southeast Regional Climate Center, Department of Geography & Environment, University of North Carolina at Chapel Hill, Chapel Hill, North Carolina, United States of America; Yunnan University, CHINA

## Abstract

Days with high diurnal temperature range (DTR) are commonly linked to morbidity and mortality, yet these health impacts are rarely connected with the underlying meteorological factors. To provide health researchers with much-needed context, we examine the climatology of DTR in the continental United States from 1950 to 2022 using the gridded ERA5 Land reanalysis archive. DTR has declined significantly over the period of record over much of the United States, with stronger signals in the east and north in summer and autumn. Extreme positive DTR values (95th percentile), which are commonly linked to morbidity, are also declining significantly. DTR declines can be ascribed to maximum temperatures increasing at a slower rate than minima, with minimum temperatures rising in response to increasing atmospheric humidity, cloud cover, and precipitation. Given that DTR trends are clearly linked to anthropogenic warming, this would imply reduced health risks, if DTR is a direct causal exposure. Because the most extreme DTR days occur under calm, low-humidity and cloud-free conditions, clinical evidence is needed to demonstrate how and why these weather conditions produce intra-day changes in exposure that are physiologically harmful.

## Introduction and context

As research on the impacts of climate on human health has become commonplace, so has the examination of potential predictors beyond the commonly used temperature and humidity metrics. Over the past decade, measures of temperature *variability* have often been examined, including diurnal temperature range (DTR), defined as the difference between the maximum and minimum temperature over the 24-hour period from midnight-to-midnight local time. Most of this research has found positive associations between DTR and poor health outcomes. Although this result is often initially hypothesized—short-term temperature extremes could strain certain organ systems—upon further examination, the relationship is less clear. Unlike air temperature, which is assumed to represent an exposure at a given time or over some time period, DTR measures a *change* in exposure. In most locations, high DTR values can occur in all seasons. Furthermore, depending on location, DTR can be correlated with maximum or minimum temperature, or both. Given the well-established relationships between temperature extremes and human health, many DTR studies either attempt to control for temperature (statistically) and/or stratify by season to unlock possible underlying relationships with DTR.

Although the consistency of results from different locations, methods, and outcome variables makes a strong case for a positive association between DTR and poor health, the clinical associations are generally lacking. There is abundant evidence on how high and low temperatures impact physiological function, but very little evidence that a relatively short-term (within 24-hour) temperature *change* produces thermal variations that strain human organ systems. For example, if a DTR ≥ 15°C is associated with increased mortality, this relationship should hold for a temperature change from 40°C to 25°C as well as from 10°C to –5°C. During a summer heat wave, when temperatures are consistently high, one might logically expect that low overnight temperatures (and therefore high DTR) would provide respite and a much-needed recovery interval rather than add to the body's environmental stress. Similarly, a warm afternoon during a winter cold wave would seemingly be welcome and physiologically beneficial. These counter-intuitive results raise the question of whether the numerous DTR studies are truly identifying relationships with temperature *change*, or if DTR is masking some other factor that is impacting the human body's response to its thermal environment [1].

From a climatological perspective, DTR is a surprisingly complex variable, as it is directly influenced by a large suite of physical variables, including cloud cover, humidity, precipitation, soil moisture, snow cover, air pressure, elevation, distance from large bodies of water, degree of urbanization and vegetation growth, and other factors [2–7]. DTR is often seasonal, with monthly patterns that vary regionally [2,6,8]. Furthermore, DTR is indirectly impacted by the atmospheric greenhouse gas concentration, and thus by both natural and anthropogenic climate variability [8–10]. As a result, human activities (urbanization, land-use change, greenhouse gas increases, etc.) have influenced the magnitude and trends of DTR.

We propose that the inherent climatological complexities of DTR need to be understood by health researchers if the community hopes to advance our interdisciplinary understanding of how and why human health is impacted by short-term temperature changes. We have thus prepared this DTR climatology primer with an emphasis on aspects of DTR that are of most interest to researchers studying human biometeorology. This study extends prior DTR climatology research by linking DTR variability and trends to their underlying meteorological drivers and by focusing on extreme DTR conditions most relevant to health studies, thereby providing a framework for interpreting DTR as a proxy for broader atmospheric processes rather than as a direct exposure. Although we selected our study area as the continental United States, similar analyses could and should be conducted in other parts of the world to provide similar background contextual information for interpreting the DTR variable within a climate-health context. We emphasize that this study is not intended to develop or apply a health exposure framework, nor to directly evaluate DTR–health relationships. Rather, our goal is to provide a climatological foundation that can inform the interpretation of DTR in epidemiological and biometeorological studies.

## Background

### Health associations with DTR

A large and growing body of literature has examined associations between DTR and human health across diverse geographic regions and populations. Most studies have focused on mortality outcomes, with consistent evidence of positive associations reported in Asia, North America, and Europe [1,11–25]. Cause-specific analyses indicate that these relationships are particularly evident for cardiovascular [1,24,26–30] and respiratory [1,16,31–34] outcomes, including both mortality and morbidity, although associations have also been identified for cerebrovascular [35–37] and other causes [38–41]. In addition to mortality, a number of studies have examined morbidity outcomes, including hospital admissions, emergency department visits, and ambulance dispatches, which likewise tend to show increased risk on days with elevated DTR [26,27,31,32,35,36,42–45].

Methodologically, most DTR–health studies employ time-series or time-stratified case-crossover designs to evaluate short-term associations between daily temperature variability and health outcomes. These approaches are typically implemented using Poisson or quasi-Poisson regression frameworks and often incorporate distributed lag structures to account for delayed effects (e.g., [14,19,46]). Most studies control for mean temperature and/or other meteorological variables and often stratify analyses by season to account for differing climatological regimes. Adjustments for long-term trends, day-of-week effects, and air pollution are also common, although model specifications vary considerably across studies.

With respect to the exposure–response relationship, most studies report a positive, approximately linear association, indicating that higher DTR is associated with increased mortality or morbidity risk. However, several studies have identified nonlinear relationships, including J-shaped or U-shaped curves, suggesting that both high and low DTR values may be associated with adverse health outcomes [16,24,27,46,47]. These findings imply that the health impacts of DTR may differ depending on the underlying atmospheric conditions, as high vs. low DTR days are driven by markedly different weather situations.

Despite the general consistency in findings across locations, outcomes, and analytical approaches, important uncertainties remain regarding the interpretation of DTR as an exposure variable. In particular, the physiological mechanisms linking short-term temperature variability to adverse health outcomes are not well established [43], and DTR is often evaluated independently of the meteorological factors that influence its magnitude. As a result, it remains unclear whether DTR represents a direct physiological stressor or serves as a proxy for a broader set of environmental conditions that may influence human health.

### Physical processes associated with DTR

DTR is the difference between temperature extremes on a given day, and the magnitude and timing of the maximum (T_max_) and minimum (T_min_) temperatures are influenced by a variety of physical processes that can interact to enhance or suppress either extreme. On a clear day, T_max_ will typically occur 2–3 hours after the time of maximum solar radiation receipt (solar noon). The time lag between maximum incoming radiation and temperature is related to the thermal responsiveness (heat capacity) of the surface type (soil, vegetation, concrete, water, etc.), which emits infrared (terrestrial) radiation, or heat, to the atmosphere. The difference between incoming solar and outgoing terrestrial radiation defines the net radiation. Although net radiation typically peaks near midday, T_max_ generally occurs later in the afternoon, after an extended period of positive net radiation and as the surface energy balance approaches zero.

Surface heat is transmitted to the atmosphere through sensible heating and by convective mixing within the daytime boundary layer. Under clear conditions, surface heating promotes turbulent mixing that entrains warmer residual air from aloft (often from the previous day) down to the surface, contributing to the daytime rise in air temperature and the evolution of T_max_. Clouds and humidity can significantly impact this timing, such that the hour of T_max_ tends to be later in the afternoon in humid climates. Water vapor is a greenhouse gas that enhances the absorption of outgoing heat from the surface, thus warming the atmosphere and delaying the rate of afternoon heat loss. Wind shifts during the day can alter temperature advection, so the transport of warmer or colder air into a region can further impact the timing of T_max_. Intra-day wind shifts are most often associated with frontal passages, in which a warm resident air mass is displaced by a colder air mass (cold front passage) or vice-versa (warm front passage). On days with cold front passages, it is possible that T_max_ could occur early in the day and T_min_ near midnight, as progressively colder air is advected southward by the winds. In mountainous regions, episodic wind events (e.g., Chinook winds) can strongly influence the magnitude of daytime temperatures and DTR as air warms while descending from higher elevations.

Similarly, T_min_ is dictated by the surface radiation balance. On clear days, Earth’s surface typically begins cooling in the mid to late afternoon. Net radiation becomes most negative shortly after sunset, when incoming shortwave radiation approaches zero and outgoing longwave radiation remains high, while T_min_ generally occurs just after sunrise following prolonged nocturnal cooling. However, the extent of nighttime cooling is greatly influenced by humidity and cloud cover (both of which retard the cooling rate), winds (strong winds mix warmer air from aloft into the boundary layer), and the number of hours of darkness. T_min_ is particularly influenced by the dew point temperature, as once the air cools to the dew point, water vapor in the air is condensed into liquid (dew or fog) which releases latent heat to the surroundings, quelling any further drop in temperature. Therefore, clear, calm nights will have lower T_min_ than overcast and/or windy nights. As with maximum temperature, T_min_ can occur at any hour of the day when there is a frontal passage and/or significant wind shifts.

Among the meteorological variables that modulate the surface radiation balance, humidity and cloud cover have a profound influence on DTR. Everything else being equal, DTR will be suppressed on an overcast day and will be largest on a clear, calm day. Days with very low DTRs are typically overcast (or foggy or extremely polluted), as there is no other mechanism to suppress both the rise in daytime temperature and the decline in nighttime temperature. Therefore, in epidemiological studies that find health responses to low DTR, it is possible that DTR is serving as a proxy for storminess, precipitation, or very poor air quality. DTR will be suppressed in midlatitude climates with warm, humid summers because the high dew point temperatures raise T_min_, and on cold and dry winter days, DTR is suppressed by the lower daytime maximum air temperatures in the cold season. Thus, the most extreme DTR values, which are the emphasis of this study, are often found on days with dry air masses in the spring and autumn, when T_max_ can approach summertime values, but the dry air (i.e., low dew point temperature) and length of night allow for a low T_min_ to be attained.

### Trends in DTR

Decreases in DTR have been documented across many parts of the globe, with some of the earliest evidence presented by Karl et al. [48]. These early findings were based primarily on station-based observational datasets, and subsequent analyses using homogenized station records have consistently confirmed widespread declines in DTR across North America and globally [49–55,56]. Reanalysis-based products, including ERA5 and related datasets, have likewise reproduced these large-scale patterns [57–59]. The widespread decline that emerged during the mid-20th century has been attributed primarily to faster increases in T_min_ relative to T_max_ [49–55]. This asymmetry in warming, where nighttime temperatures rise more rapidly than daytime temperatures, has been widely noted in recent decades [49,50,60,61] and has contributed to a long-term global reduction in DTR [55,56,62,63]. Some portion of this asymmetry has also been linked to non-climatic influences, including urbanization surrounding weather stations (a process not explicitly represented in most reanalysis datasets, such as ERA5-Land), which can elevate nighttime temperatures and artificially suppress DTR. Prior to 1950, DTR trends are uncertain because of sparse and uneven station records [56], but longer-term data suggest a shift from increasing to decreasing DTR beginning around 1950, followed by a flattening of the global trend between 1979 and 2004 as increases in T_max_ began matching those of T_min_ [52]. Recent studies note a renewed increase in DTR in parts of the world (including North America) linked to reductions in aerosols and shifts in cloud cover and precipitation [64–66]. With respect to future trends in DTR, climate model projections remain mixed and depend strongly on emissions pathways and associated regional climate processes. A recent study by Wang et al. [67] suggests a continued decline in DTR under intermediate and high emissions scenarios, particularly over parts of North America, with a shift towards increasing DTR under low emissions scenarios.

### Materials and methods

Hourly ERA5-Land reanalysis data for the continental United States (CONUS) were accessed through Google Earth Engine for the period January 1950 through December 2022. ERA5-Land is a high-resolution dataset that provides a consistent record of land surface variables over several decades [68]. Although reanalysis datasets are model-based, they are widely used to assess long-term spatial and temporal climate patterns due to their consistency and spatial completeness [57–59]. DTR, particularly its extreme values, can be sensitive to land surface processes and local-scale variability that may not be fully resolved in reanalysis products. However, ERA5-Land has been shown to reproduce observed temperature variability and long-term trends consistent with station-based datasets [57–59]. In addition, comparisons with station observations at representative locations indicate that the seasonal structure of DTR is well captured, supporting the use of ERA5-Land for regional-scale climatological analysis (see S1 Fig). ERA5-Land improves upon the spatial resolution of the original ERA5 dataset by rerunning the land component of the ECMWF ERA5 reanalysis, offering data at a resolution of approximately 9 km. Using the hourly data, we extracted 2-m air temperatures to determine T_max_ and T_min_ values for each day (converted from UTC to local time). We also obtained the 2-m dew point temperature and surface pressure at the time of T_max_ and T_min_ and calculated the number of hours of measurable precipitation (≥ 0.127 mm) per day. These variables were selected to help determine those meteorological factors that influence the spatio-temporal characteristics and variability of DTR.

Given the large spatial extent of our study area, the native 9 km resolution was finer than necessary for a regional-scale analysis. To better capture broad spatial patterns, we resampled the data by aggregating the 9 km cells to a coarser 150 km resolution, resulting in a spatial field of 480 grid cells. Aggregating to this scale facilitates clearer regional comparisons, aligns with the spatial scale of many climate-health applications, and reduces the influence of localized variability that may obscure larger-scale signals. For each 150 km cell, we calculated daily DTR values and summarized their distribution by month over the period of record. Initial examination of monthly patterns and trends revealed consistent seasonal patterns, so we instead aggregated the daily results by meteorological seasons (winter: DJF; spring: MAM; summer: JJA; autumn: SON). For analysis and visualization purposes, we encoded each grid cell as a node (represented as the centroid of the cell).

Because extreme DTR values have been linked to increased risk of adverse health outcomes, we focused on the upper tail of the DTR distribution. Specifically, we identified the 95th percentile DTR threshold at each grid node over the period of record and then counted the number of days in each season-year when this threshold was equaled or exceeded. To assess long-term trends in extreme DTR, we performed linear regression on the seasonal exceedance counts and evaluated statistical significance at the 95% confidence level. We report standardized regression slopes (β) to enable comparison of trend magnitudes across regions that differ in the variability of seasonal exceedance counts. Standardization provides a scale-independent measure of change, allowing spatial differences in the magnitude of trends to be compared directly. To account for temporal autocorrelation, trend models were fit using generalized least squares (GLS) with a first-order autoregressive [AR(1)] error structure. Mapped effect sizes were expressed as standardized regression coefficients obtained by fitting the GLS model to standardized predictor and response variables at each grid node. Statistical significance of the standardized coefficients was taken directly from the GLS model output. To evaluate the robustness of the results, we repeated the exceedance analysis using alternative extreme DTR thresholds based on the 97.5th and 99th percentiles. Results using these higher thresholds were consistent with those obtained using the 95th percentile, indicating that our conclusions are not sensitive to the specific definition of extreme DTR. The 95th percentile is also a common threshold choice in DTR-health research [26,27,30,39,40].

Climate variables are spatially correlated to varying degrees. Although our primary purpose is to explore general patterns of DTR, we are also interested in the strength of any long-term trends. To account for spatial autocorrelation across grid nodes and the true degrees of freedom in the spatial field, we calculated the False Discovery Rate (FDR; [69]), which results in an adjustment to the critical p value. We calculated the FDR based on the sample of 480 grid nodes and used a type I error rate of 0.10, which is a commonly applied threshold in climatological field significance testing [70].

## Results

### Mean DTR by season

**Fig 1:**
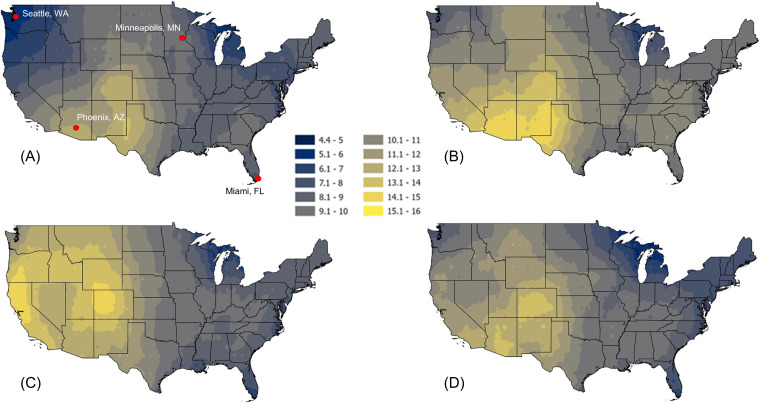
Mean DTR (°C) for (A) winter, (B) spring, (C) summer, and (D) autumn. Locations described in S1 Fig are shown on map (A). Base map from ESRI (VGIN, Esri, HERE, Garmin, FAO, NOAA, USGS, EPA, NPS; url: https://www.arcgis.com/home/item.html?id=979c6cc89af9449cbeb5342a439c6a76).

The spatial patterns of mean DTR are broadly consistent with prior climatological studies; here, they are presented to provide context for subsequent analyses of trends, extremes, and underlying meteorological drivers. Fundamentally, DTR is largest at dry (low humidity) locations and/or during dry periods. Mean DTR exhibits distinct seasonal and geographic patterns across the CONUS. During winter, the lowest values occur in the Pacific Northwest, Great Lakes, and portions of the Northeast, while the highest values are concentrated in the Southwest, including southern Arizona, eastern New Mexico, western Texas, and Colorado (Fig 1a). In spring, DTR increases across most of the CONUS, with persistent minima in the Pacific Northwest and Great Lakes and an expanded region of high values across the Southwest (Fig 1b). Summer features the broadest spatial contrast, with maximum DTR values across the Intermountain West and West Coast and minimum values across the eastern CONUS, particularly the Great Lakes, mid-Atlantic, and northern Gulf Coast (Fig 1c). In autumn, DTR decreases across much of the CONUS but remains relatively high in the Southwest and Intermountain West, while the lowest values persist across the Great Lakes, Northeast, and along the East Coast (Fig 1d).

The influence of dry air on DTR is clear in examining the seasonal signals at four representative locations derived from station observations (S1 Fig). The mean monthly DTR in the desert of Phoenix, AZ, is substantially larger than in subtropical warm and humid Miami, FL. The spring and autumn peaks in Phoenix and Minneapolis, MN (mid-latitude continental interior), and the summer peak in Seattle, WA (temperate maritime), occur during months with lower cloud cover and precipitation. These station-based records exhibit seasonal DTR patterns consistent with those derived from ERA5-Land (Fig 1), providing an independent point of comparison that supports the accuracy of the reanalysis data.

### Trends in mean and extreme DTR by season

**Fig 2:**
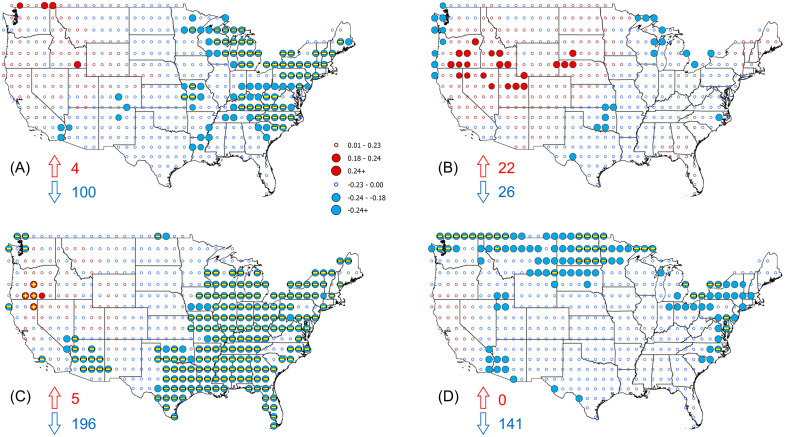
Trend in mean DTR for (A) winter, (B) spring, (C) summer, and (D) autumn. Red (blue) circles indicate increasing (decreasing) trends. Filled (open) circles denote statistically significant (nonsignificant) trends where α = 0.05. Circle size is proportional to the standardized regression slope magnitude, such that larger (smaller) circles correspond to larger (smaller) trends. The number of grid nodes with statistically significant trends is provided for each season next to the up-arrow (increasing trend) and down-arrow (decreasing trend). Plus and minus signs indicate those grid nodes that passed the FDR test that accounts for spatial autocorrelation. Base map from ESRI (VGIN, Esri, HERE, Garmin, FAO, NOAA, USGS, EPA, NPS; url: https://www.arcgis.com/home/item.html?id=979c6cc89af9449cbeb5342a439c6a76).

While long-term declines in DTR associated with asymmetric warming are well documented, the seasonal structure of these trends, their relationship to underlying meteorological drivers, and their behavior in the upper tail of the distribution remain less well characterized, particularly in a framework relevant to health research. Across seasons, DTR trends are characterized by widespread declines across much of the eastern CONUS and more variable, often nonsignificant changes in the western CONUS. In winter (Fig 2a), statistically significant decreases are concentrated across the Great Lakes, Northeast, mid-Atlantic, and Mississippi River Valley, with additional declines in portions of the Southwest. In contrast, the western CONUS exhibits small and mostly nonsignificant increases. Spring exhibits fewer significant trends (Fig 2b). DTR generally decreases across much of the eastern CONUS and parts of the West, though statistically significant changes are limited and scattered, particularly across the Great Lakes, Ozarks, and Pacific Northwest. Summer shows the most extensive and spatially coherent declines (Fig 2c), especially across the eastern CONUS and parts of the Southwest. In contrast, trends in the western CONUS are generally weak and nonsignificant. In autumn, DTR declines across much of the CONUS (Fig 2d), with significant decreases concentrated across the northern tier, mid-Atlantic, and portions of the Southwest.

Because previous studies have shown that health impacts tend to occur on days with high DTR, we next examine trends in the seasonal counts of DTR values exceeding the 95th percentile.

**Fig 3:**
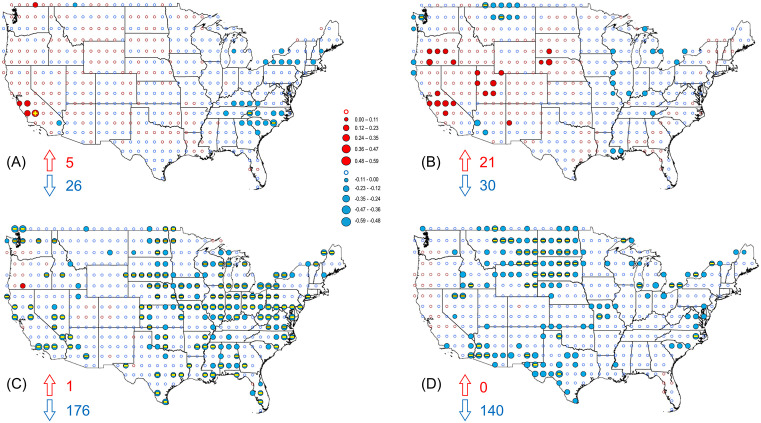
Same as Fig 2 but for the trend in extreme DTR for (A) winter, (B) spring, (C) summer, and (D) autumn. Base map from ESRI (VGIN, Esri, HERE, Garmin, FAO, NOAA, USGS, EPA, NPS; url: https://www.arcgis.com/home/item.html?id=979c6cc89af9449cbeb5342a439c6a76).

Patterns in extreme DTR largely mirror those observed for mean DTR, with the most pronounced and widespread declines occurring in summer and autumn. In winter (Fig 3a), declines in extreme DTR are observed across portions of the Northeast and Southeast, with more limited and scattered changes elsewhere. Spring shows relatively few significant trends (Fig 3b), with only isolated increases and decreases across the Midwest, Great Lakes, and western CONUS. In contrast, summer exhibits widespread and significant declines in extreme DTR (Fig 3c), particularly across the eastern CONUS and extending into parts of the High Plains and Southwest. Autumn also shows numerous and often large decreases (Fig 3d), especially across the northern tier of the CONUS.

### Trends in maximum and minimum temperatures

**Fig 4:**
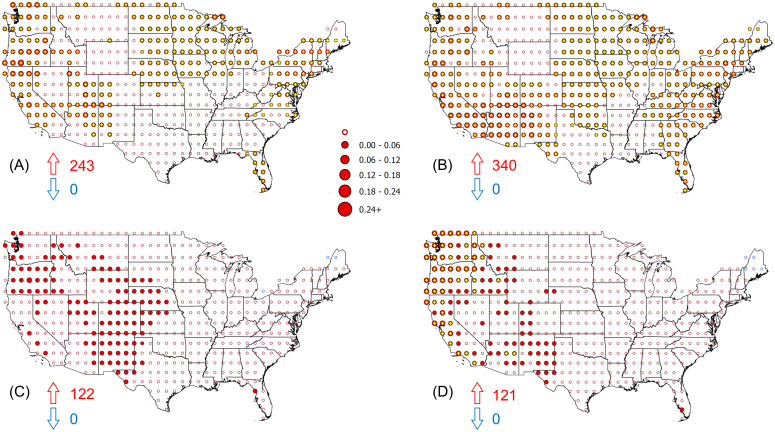
Trends in (A) maximum and (B) minimum temperatures during winter, and (C) maximum and (D) minimum temperatures during spring. Plus and minus signs indicate those grid nodes that passed the FDR test that accounts for spatial autocorrelation. Base map from ESRI (VGIN, Esri, HERE, Garmin, FAO, NOAA, USGS, EPA, NPS; url: https://www.arcgis.com/home/item.html?id=979c6cc89af9449cbeb5342a439c6a76).

To evaluate the potential drivers of observed DTR trends, we next examine seasonal trends in T_max_ and T_min_. We also calculate correlation coefficients between DTR and T_max_ and T_min_ to characterize how the strength and direction of these relationships vary seasonally and spatially (see Supporting Information). In winter, increasing trends in many parts of the CONUS are noted for both T_max_ and T_min_ (Fig 4a and 4b). However, increases in T_min_ are generally larger in magnitude than those in T_max_, resulting in declining DTR. Nearly all grid nodes show strong, statistically significant negative correlations between T_min_ and DTR, indicating that increases in nighttime temperatures strongly reduce DTR (S2 Fig). Correlations between DTR and T_max_ are mostly positive, but fewer are statistically significant, and most are near zero.

In spring, T_max_ and T_min_ are also increasing across much of the western CONUS (Fig 4c and 4d). Correlation coefficients are positive there, with the strongest associations between DTR and T_max_ (S3 Fig). Across the eastern CONUS, where spring DTR has declined, T_min_ is increasing more rapidly than T_max_, and correlations between T_min_ and DTR are strongly negative, similar to the winter pattern.

**Fig 5:**
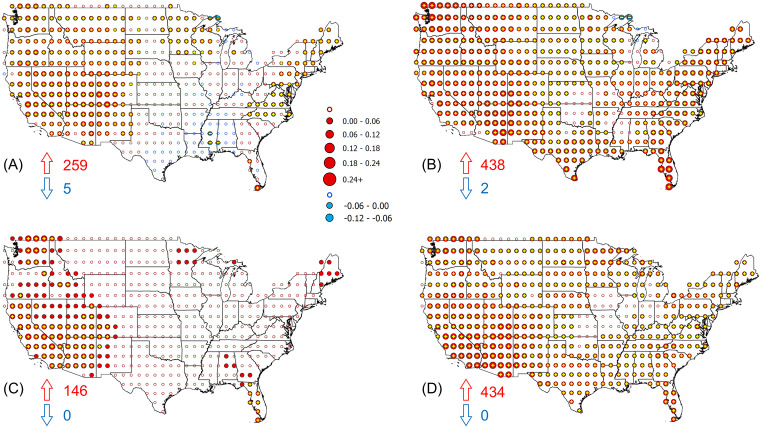
Same as Fig 4 but for (A,B) summer and (C,D) autumn. Base map from ESRI (VGIN, Esri, HERE, Garmin, FAO, NOAA, USGS, EPA, NPS; url: https://www.arcgis.com/home/item.html?id=979c6cc89af9449cbeb5342a439c6a76).

Summer shows a more complex spatial structure (Fig 5a and 5b). T_max_ and T_min_ are increasing across many regions, including the western CONUS and the Northeast, but decreasing trends in T_max_ are observed across portions of the southern CONUS and Midwest, particularly in Mississippi and Alabama, corresponding to the well-documented “Warming Hole” [71]. This region also exhibits increases in T_min_, though trends are smaller than those in the Northeast. As in other seasons, correlations between DTR and T_max_ are generally positive, while correlations with T_min_ are mostly negative (S4 Fig). The largest decreases in summer DTR occur where T_max_ is declining and T_min_ is increasing, particularly across the eastern CONUS. In contrast, across much of the West, T_max_ and T_min_ are increasing at similar rates, resulting in minimal or nonsignificant summer DTR trends.

In autumn, T_max_ is increasing across most grid nodes, although significant trends are limited to the western CONUS and Florida (Fig 5c). T_min_ is also increasing, with larger magnitudes and widespread statistical significance (Fig 5d). These combined associations align with the widespread significant decreases in DTR observed in autumn, particularly across the northern tier of the CONUS. Correlations between DTR and T_min_ are strongly negative, consistent with patterns found elsewhere. Correlations with T_max_ are also negative across many areas in autumn (S5 Fig).

### Climatological composites of extreme DTR days

**Fig 6:**
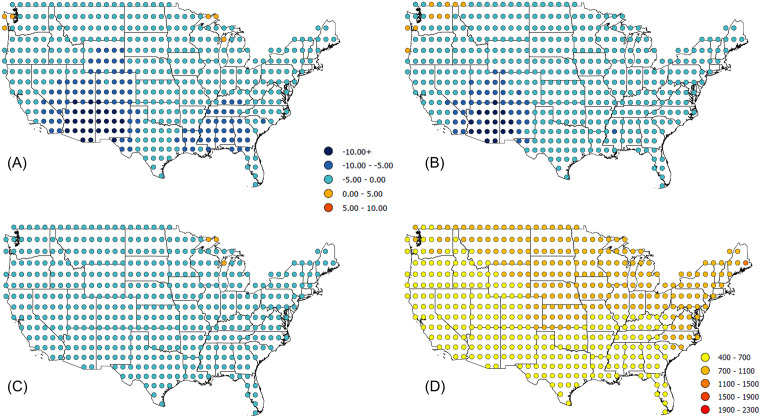
Summer anomalies in (A) dew point temperature at time of maximum temperature, (B) dew point temperature at time of minimum temperature, (C) precipitation frequency (hours), and (D) surface pressure (Pa) at the time of minimum temperature. Anomalies are the difference in the values of each variable on high DTR days (DTR ≥ 95th percentile value) and the seasonal mean of that variable at each grid node. Base map from ESRI (VGIN, Esri, HERE, Garmin, FAO, NOAA, USGS, EPA, NPS; url: https://www.arcgis.com/home/item.html?id=979c6cc89af9449cbeb5342a439c6a76).

To complement the physical interpretation of high DTR conditions, we constructed composite anomaly fields of selected meteorological variables on days with extreme DTR (≥95th percentile) minus the nodal mean value. Across seasons, extreme DTR days are characterized by systematically lower dew point temperatures, particularly at the time of T_min_, indicating drier atmospheric conditions. These differences are especially pronounced in summer (Fig 6), when large negative dew point anomalies coincide with widespread reductions in precipitation frequency, consistent with reduced cloud cover on high DTR days. Surface pressure is uniformly higher on extreme DTR days, reflecting the presence of synoptic-scale high-pressure systems associated with subsidence and clear skies. These results support the underlying physical arguments that extreme DTR events occur under a consistent set of meteorological conditions—dry days with low humidity, low cloud cover, and high pressure. (Composite anomaly maps for the other seasons are provided in S6–S8 Figs).

### Trends in dew point temperatures and precipitation

To further assess the meteorological controls shaping DTR trends, we examined seasonal trends in dew point temperature at the time of daily T_max_ and T_min_, along with trends in precipitation frequency. These variables provide insight into how atmospheric moisture and humidity modulate short-term temperature variability.

**Fig 7:**
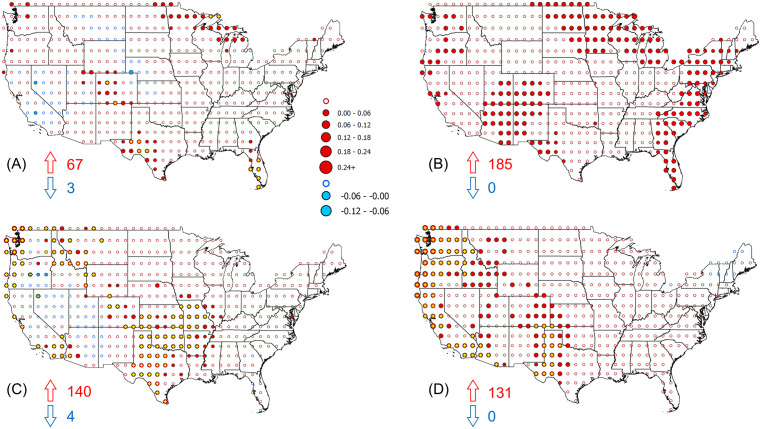
Trends in dew point temperature at the time of (A) maximum and (B) minimum temperatures during winter, and (C) maximum and (D) minimum temperatures during spring. Plus and minus signs indicate those grid nodes that passed the FDR test that accounts for spatial autocorrelation. Base map from ESRI (VGIN, Esri, HERE, Garmin, FAO, NOAA, USGS, EPA, NPS; url: https://www.arcgis.com/home/item.html?id=979c6cc89af9449cbeb5342a439c6a76).

In winter, dew point temperatures have increased at both T_max_ and T_min_, with larger increases occurring at T_min_ (Fig 7a and 7b). These changes are consistent with observed increases in winter T_min_ and the associated decline in DTR. Spring exhibits a similar pattern, with increasing dew points at both T_max_ and T_min_, the latter exhibiting larger increases (Fig 7c and 7d). These conditions correspond with reductions in DTR across the central Plains and Pacific Northwest.

**Fig 8:**
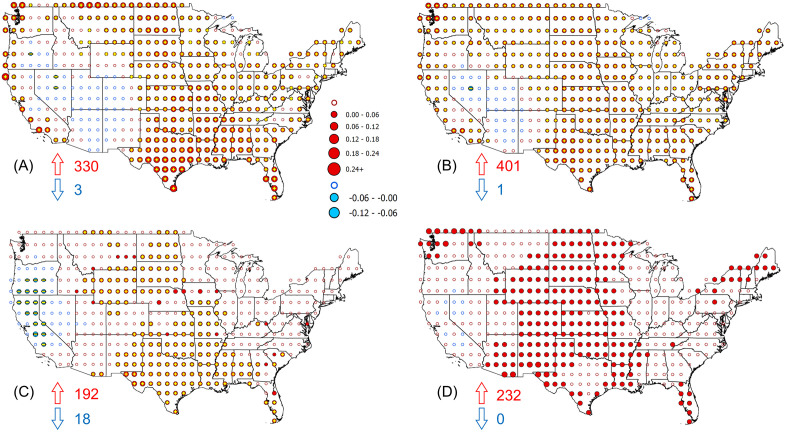
Same as Fig 7 but for (A,B) summer and (C,D) autumn. Base map from ESRI (VGIN, Esri, HERE, Garmin, FAO, NOAA, USGS, EPA, NPS; url: https://www.arcgis.com/home/item.html?id=979c6cc89af9449cbeb5342a439c6a76).

Summer shows widespread and significant increases in dew point at both T_max_ and T_min_ (Fig 8a and 8b). In the “Warming Hole” region of the southeastern and south-central CONUS, increasing dew point appears to contribute to both increasing T_min_ and suppressed T_max_, resulting in declining DTR. Autumn also exhibits increasing dew point trends, except in the western CONUS (Fig 8c and 8d). In this region, decreasing dew point at the time of T_max_ paired with increases at T_min_ reflects daytime drying and nighttime moistening patterns that correspond with weak or positive DTR trends. Elsewhere, the seasonal moistening in autumn aligns with modest trends in T_max_, widespread increases in T_min_, and declines in DTR across the northern tier and parts of the southern tier of the CONUS. Both summer and autumn also show significant decreases in extreme DTR values, which align more strongly with changes in T_min_ than T_max_, as indicated by stronger correlations between DTR and T_min_. Where T_max_ exhibits little trend, the decline in extreme DTR values appears tied to nighttime moisture increases and rising temperatures.

Analysis of precipitation frequency supports these relationships. Winter precipitation frequency trends are generally weak across the eastern CONUS, though mostly positive and consistent with declining DTR, whereas decreasing trends in the Northwest align with localized increases in DTR (S9 Fig). In spring, decreasing precipitation frequency in parts of the western CONUS are consistent with increasing DTR, while significant increases across the interior CONUS correspond with increasing dew point and declining DTR. Summer exhibits strong regional contrasts. Decreasing precipitation frequency in the West corresponds with daytime drying and increasing DTR, whereas significant increases across the eastern CONUS coincide with elevated dew point and decreasing DTR. In autumn, precipitation frequency increases across the High Plains, Great Lakes, and lower Mississippi River Valley, supporting decreases in both mean DTR and counts of extreme DTR values, while decreases in precipitation frequency are noted in the western CONUS.

## Discussion

Although DTR is a very simple variable to calculate, its role as an exposure variable in biometeorology and epidemiology is quite complex. The underlying physical processes that control the variation in DTR over time and space may not be intuitive to health researchers, as DTR is the result of a complex interplay of meteorological factors, especially humidity, cloud cover, and precipitation. For example, since DTR is calculated from T_max_ and T_min_, one might assume that its variation is equally correlated with both, but that is not necessarily the case. In some locations and months, DTR can be completely uncorrelated with either variable (e.g., [2,43], S2–S5 Figs). We propose that developing a physiological understanding of how and why DTR impacts human health requires a parallel understanding of the physical drivers of this variable.

Our key messages are:

1. DTR is not inherently an exposure variable; rather it represents a *change* in exposure.
2. DTR is *declining* across the United States. Although both T_max_ and T_min_ are rising in most places, the increase in minima is outpacing the rise in maxima.
3. The declines in DTR are associated with increasingly humid conditions, especially in the summer and autumn.
4. The frequency of extreme positive DTR days is likewise decreasing.

With respect to message 1, most studies that have identified positive associations between DTR and health simultaneously control for temperature in some manner [22,31,27,32,46]. Thus, if the effects of temperature have been fully eliminated, affected individuals are facing difficulties coping with large temperature changes over 24 hours. However, people routinely encounter very rapid changes over the course of a typical day, such as when entering or leaving an air-conditioned building on a hot and humid afternoon. If there is a direct health impact of these changes, it is likely manifested through the respiratory system, which is fully exposed to ambient conditions (unlike the skin, which is protected). Some research has uncovered direct linkages between high DTR (while controlling for temperature) and respiratory illness [31,32,44,46,72]. Furthermore, a body of research exists suggesting that a lack of short-term acclimatization can exert physiological strain on organ systems, and that transitions from warm-humid to cold-dry climates provide the greatest respiratory challenge to acclimatization [73–75]. This notion has not been investigated in the context of DTR but has the potential for improving our nascent understanding of the physiological linkages between DTR and health.

In DTR studies, a common motivation is that anthropogenic climate change is resulting in more extremes, thus increasing disease risk. With respect to DTR, in general, this is fundamentally incorrect. We find that, since 1950, DTR has been declining significantly across the continental United States. Similar DTR trends have been observed in many other locations [9,51,55]. The primary reason is that minimum temperatures are increasing faster than maxima. As the planet warms and more moisture is evaporated or transpired into the air, dew point temperatures increase. On calm and clear nights, the dew point temperature is a good proxy for the minimum temperature. Furthermore, increasing cloud cover from a moister atmosphere will constrain the rate of nighttime cooling. In addition, the urban heat island effect, in which buildings and other structures absorb daytime heat and release it at night, has been shown to raise minimum temperatures and reduce DTR in station-based studies [76, 77] (although such effects are not explicitly resolved in the ERA5-Land dataset used here). Thus, urbanization raises minimum temperatures and reduces DTR. Over time, the net effect is that nighttime and early morning minimum temperatures have been rising, so DTR has been declining.

Most DTR-health research finds linear or J-shaped relationships between DTR and poor health outcomes. In general, the strongest responses occur at extreme high values of DTR—days with large differences between T_max_ and T_min_. This common result motivated the focus of this study on the subset of days with the highest DTR values. As with DTR in general, we find that the occurrence of extreme DTR (95th percentile) is decreasing across the United States. If one believes in a causal linkage between high DTR days and poor health outcomes, this implies that climate change should reduce the health risk arising from exposure to extremely high DTRs.

Specifically, there is strong evidence of declining trends in every season but spring, and they are generally concentrated in the eastern half of the United States. The decline in extremes can be explained by the increasing dew point temperature at most of those grid nodes with significant trends. Higher nighttime and early morning dew points effectively raise the minimum temperature floor on dry days with calm and clear nights. In the eastern United States, these days are rare in the summer given the humid climate and frequent cloud cover. In winter, days with dry air masses are more common, but there is still a trend in declining extreme DTR days. Presumably the positive dew point trends at the time of T_min_ implies that the coldest winter nights have warmed over time as the humidity has increased. In autumn, the DTR declines tend to be concentrated among the northern grid nodes, especially along the Great Lakes, Upper Midwest, and Northern Plains. These locations experience the earliest onset of winter, and we speculate that the cold air outbreaks from Canada have become somewhat warmer and more humid over time on these coldest days [78, 79]. The spring results proved to be the least conclusive and interesting, with no obvious spatial pattern and generally weaker trends.

A much smaller group of studies have uncovered U-shaped relationships, indicating poor outcomes on days with very low DTR as well [24,27,46]. Aside from polar climates, the only situation in which DTR can be small are days with thick cloud cover, persistent fog, or extremely poor air quality. Often, these low DTR days will also have steady precipitation. Thus, for those places where low DTR is associated with negative health outcomes, the underlying physiological mechanism must be entirely different from the more common high DTR impacts.

We wish to emphasize that our goal in this research is not to provide the definitive climatology of DTR in the continental United States, but to contextualize DTR so that researchers who study climate and health might better interpret and understand DTR-health relationships. A more climatologically-oriented study could utilize a finer grid mesh or compare DTR patterns in different gridded networks (such as PRISM). We also acknowledge that reanalysis datasets such as ERA5-Land may not fully resolve local-scale processes influencing DTR, particularly for extreme values that are sensitive to land surface heterogeneity and boundary layer dynamics. Although this may introduce some uncertainty at finer spatial scales, our focus was on large-scale spatial patterns and long-term trends, features that are well represented in both reanalysis and station-based datasets. We assumed a linear fit to examine trends, but we fully acknowledge that trends may be nonlinear and that break points may exist between climatic regimes. Although we specifically examined humidity to explain some of the DTR variability, we fully acknowledge that numerous other factors may also be responsible, including urbanization, air quality, cloud cover, amongst others. These variables are not explicitly included in the ERA-5 Land data archive. Furthermore, we recognize that there are more sophisticated methods to account for the spatial correlation present in our data, such as by explicitly modeling the spatial covariance, but given our purposes we believe the FDR correction provides a reasonable adjustment for this issue.

## Conclusions

We estimate that over 100 studies have been published in the refereed scientific and medical literature linking DTR to human health. Most have found associations between days with high DTR values and elevated morbidity and mortality. Despite this research emphasis, a clear physiological understanding of the reason for these correlations is lacking. We suggest that this arises from a misunderstanding of the atmospheric processes affecting DTR coupled with a paucity of clinical studies focused on how the human body responds to shorter term *changes* in temperature.

Our findings indicate that extreme DTR events arise from a consistent set of synoptic conditions rather than from extreme events such as heat waves or cold spells. Consequently, DTR should be interpreted as a proxy for a broader range of weather conditions rather than as an isolated stressor. From a climatological perspective, in general, days with high DTR tend to be clear with low humidity. Clear skies allow for both high T_max_ and low T_min_, as heat from the surface can readily radiate through the atmosphere to space when clouds are lacking, resulting in cool mornings. Under more humid conditions, not only are clouds more likely, but the nighttime cooling rate is mitigated by the higher dew point temperatures. Therefore, the weather on high DTR days will tend to be clear and dry (low humidity) with warm or hot afternoons and cool conditions in the early morning.

From a physiological perspective, one must ask why negative health impacts increase on what most people consider to be among the more pleasant days. If the statistical models are effectively and completely controlling for temperature, then these results are not related to exposure to either hot afternoons or cold mornings specifically, but to the body's ability to adjust to temperature changes over the course of 24 hours. This is complicated by the fact the people routinely and comfortably transition from indoor and outdoor environments with different temperatures in seconds with presumably little to no ill health effects.

Our climatological analysis in the continental United States has shown that DTR is decreasing at a statistically significant rate since 1950 across most of the country. This long-term decline is driven by T_min_ increasing at a faster rate than T_max_ at most locations. Given that dew point temperatures at the time of T_min_ are also increasing, it is likely that this long-term trend is at least partly related to an increasingly humid atmosphere, which makes cloud cover more likely and low nighttime minimum temperatures less likely. Importantly from a human health perspective, these trends apply on those days with the highest DTR values, especially in the eastern and northern part of the country, and in summer and autumn seasons. This would suggest the potential for reduced health risk associated with high DTR, although this interpretation depends on the extent to which DTR itself represents a causal exposure rather than a proxy for other meteorological conditions. Because DTR may serve as a proxy for broader meteorological conditions, trends in DTR do not translate directly into trends in health risk. The observed decline in DTR is largely driven by increases in nighttime temperatures and atmospheric moisture, both of which have independent and potentially opposing effects on human health. As a result, declining DTR may reflect a shift in the underlying atmospheric drivers of exposure rather than a simple reduction in risk.

By framing DTR within its physical, seasonal, and geographical contexts, we provide a foundation for health researchers to more accurately attribute outcomes, better control for confounding meteorological influences, and refine interpretations of the physiological relevance of DTR. This perspective shifts the role of DTR in biometeorological research from a “black-box” indicator of thermal stress to a diagnostic tool for understanding the integrated nature of weather, climate, and health. Ultimately, targeted climatological and clinical studies are needed to unravel the mystery of DTR-health linkages. These would include a careful, patient-level analysis of the diseases and conditions on high DTR days, along with clinical research examining physiological changes to individuals over varied temperature ranges in 24 hours.

## Supporting information

S1 Fig Monthly mean DTR derived from station observations at select locations shown in Fig 1a, including Phoenix, AZ (PHX), Miami, FL (MIA), Minneapolis, MN (MSP), and Seattle, WA (SEA). (TIF)

S2 Fig Correlation coefficients between (A) maximum and (B) minimum temperatures and diurnal temperature range during winter. The number of grid nodes with statistically significant correlations is provided for each season next to the plus sign (positive correlations) and minus sign (negative correlations). Base map from ESRI (VGIN, Esri, HERE, Garmin, FAO, NOAA, USGS, EPA, NPS; url: https://www.arcgis.com/home/item.html?id=979c6cc89af9449cbeb5342a439c6a76).
(TIF)

S3 Fig Same as S2 Fig, but for spring. Base map from ESRI (VGIN, Esri, HERE, Garmin, FAO, NOAA, USGS, EPA, NPS; url: https://www.arcgis.com/home/item.html?id=979c6cc89af9449cbeb5342a439c6a76).
(TIF)

S4 Fig Same as S2 Fig, but for summer. Base map from ESRI (VGIN, Esri, HERE, Garmin, FAO, NOAA, USGS, EPA, NPS; url: https://www.arcgis.com/home/item.html?id=979c6cc89af9449cbeb5342a439c6a76).
(TIF)

S5 Fig Same as S2 Fig, but for autumn. Base map from ESRI (VGIN, Esri, HERE, Garmin, FAO, NOAA, USGS, EPA, NPS; url: https://www.arcgis.com/home/item.html?id=979c6cc89af9449cbeb5342a439c6a76).
(TIF)

S6 Fig Same as Fig 6 but for winter. Base map from ESRI (VGIN, Esri, HERE, Garmin, FAO, NOAA, USGS, EPA, NPS; url: https://www.arcgis.com/home/item.html?id=979c6cc89af9449cbeb5342a439c6a76).
(TIF)

S7 Fig Same as Fig 6 but for spring. Base map from ESRI (VGIN, Esri, HERE, Garmin, FAO, NOAA, USGS, EPA, NPS; url: https://www.arcgis.com/home/item.html?id=979c6cc89af9449cbeb5342a439c6a76).
(TIF)

S8 Fig Same as Fig 6 but for autumn. Base map from ESRI (VGIN, Esri, HERE, Garmin, FAO, NOAA, USGS, EPA, NPS; url: https://www.arcgis.com/home/item.html?id=979c6cc89af9449cbeb5342a439c6a76).
(TIF)

S9 Fig Trends in precipitation frequency for (A) winter, (B) spring, (C) summer, and (D) autumn. Plus and minus signs indicate those grid nodes that passed the FDR test that accounts for spatial autocorrelation. Base map from ESRI (VGIN, Esri, HERE, Garmin, FAO, NOAA, USGS, EPA, NPS; url: https://www.arcgis.com/home/item.html?id=979c6cc89af9449cbeb5342a439c6a76).
(TIF)
