# Supplementary figures and images for: Diurnal temperature range: A climatological primer for health researchers
Source: PLoS One. 2026 Jul 7;21(7):e0352866. doi: 10.1371/journal.pone.0352866 (PMC13340791; doi:10.1371/journal.pone.0352866)

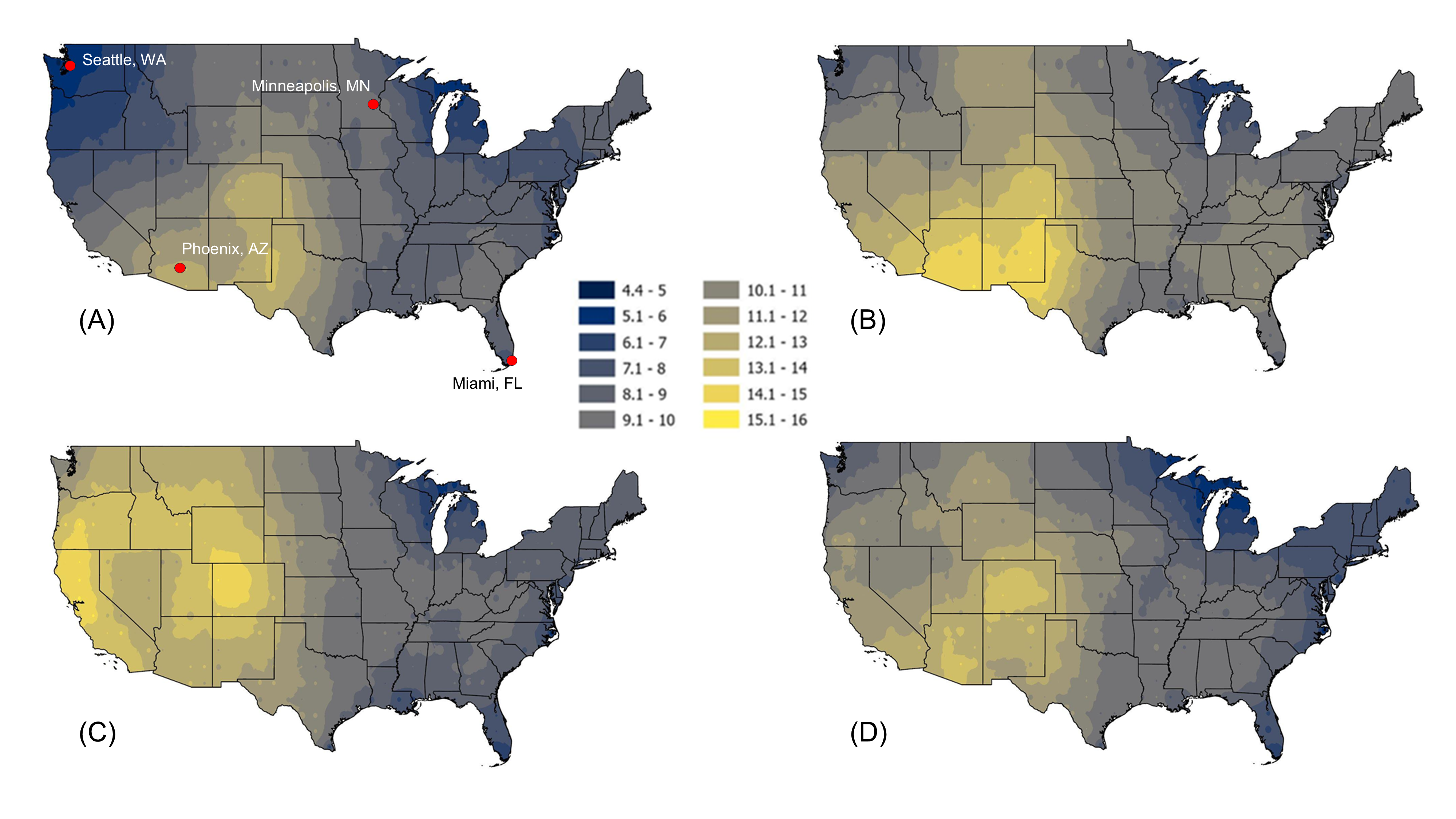

Supplement: S1 Fig — (TIF) [file pone.0352866.s001.tif]

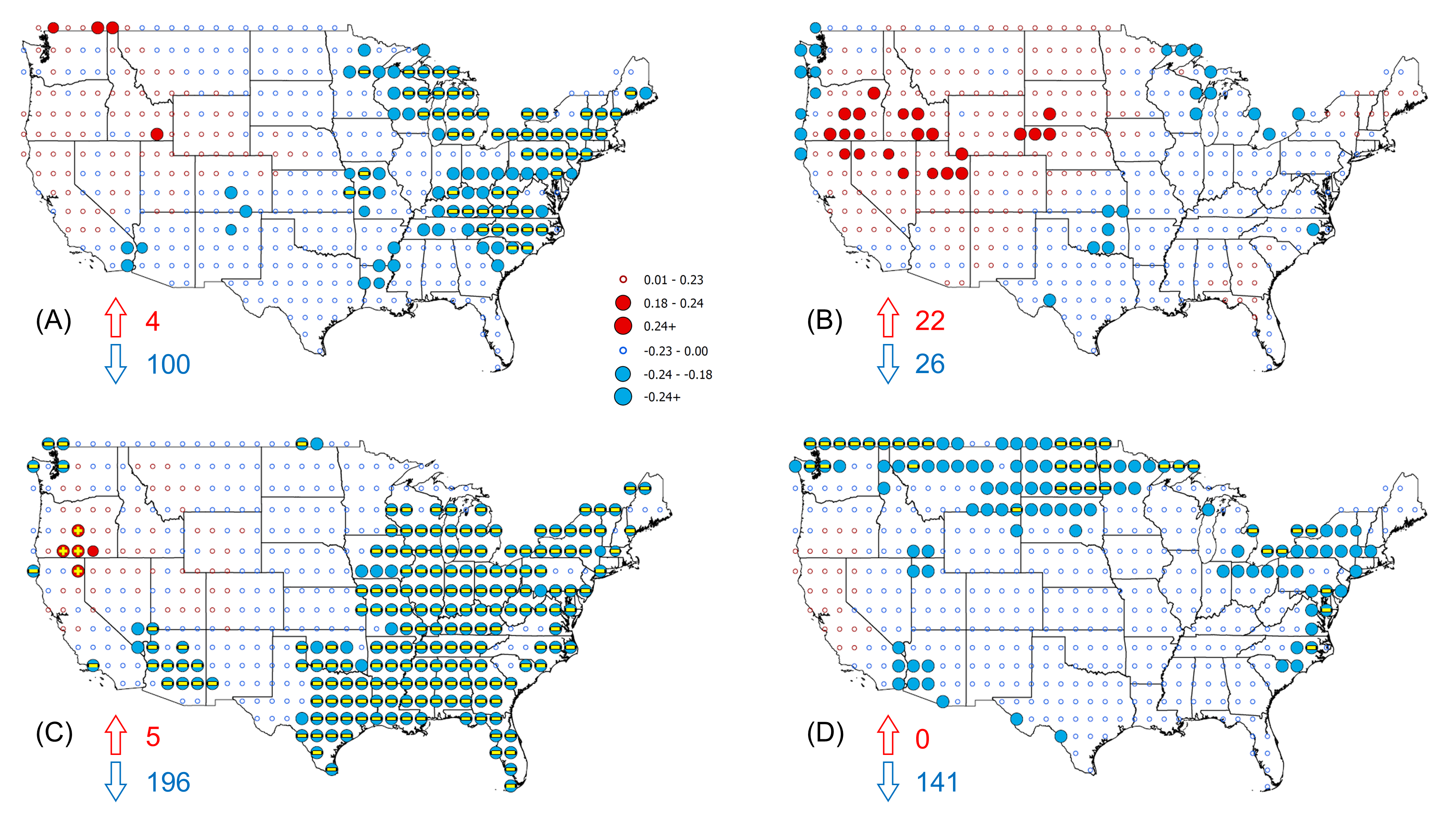

Supplement: S2 Fig — The number of grid nodes with statistically significant correlations is provided for each season next to the plus sign (positive correlations) and minus sign (negative correlations). Base map from ESRI (VGIN, Esri, HERE, Garmin, FAO, NOAA, USGS, EPA, NPS; url: https://www.arcgis.com/home/item.html?id=979c6cc89af9449cbeb5342a439c6a76). (TIF) [file pone.0352866.s002.tif]

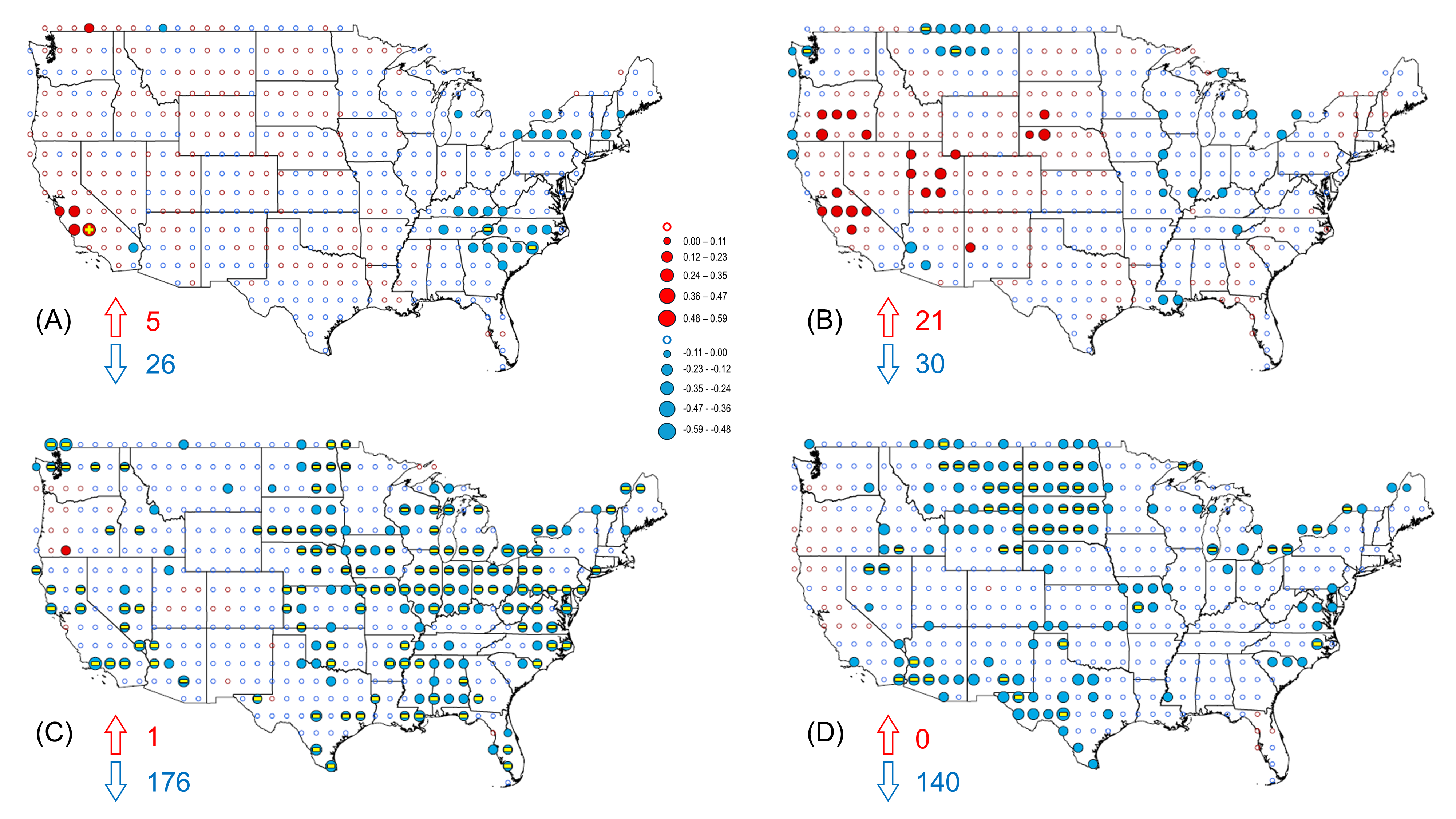

Supplement: S3 Fig — Base map from ESRI (VGIN, Esri, HERE, Garmin, FAO, NOAA, USGS, EPA, NPS; url: https://www.arcgis.com/home/item.html?id=979c6cc89af9449cbeb5342a439c6a76). (TIF) [file pone.0352866.s003.tif]

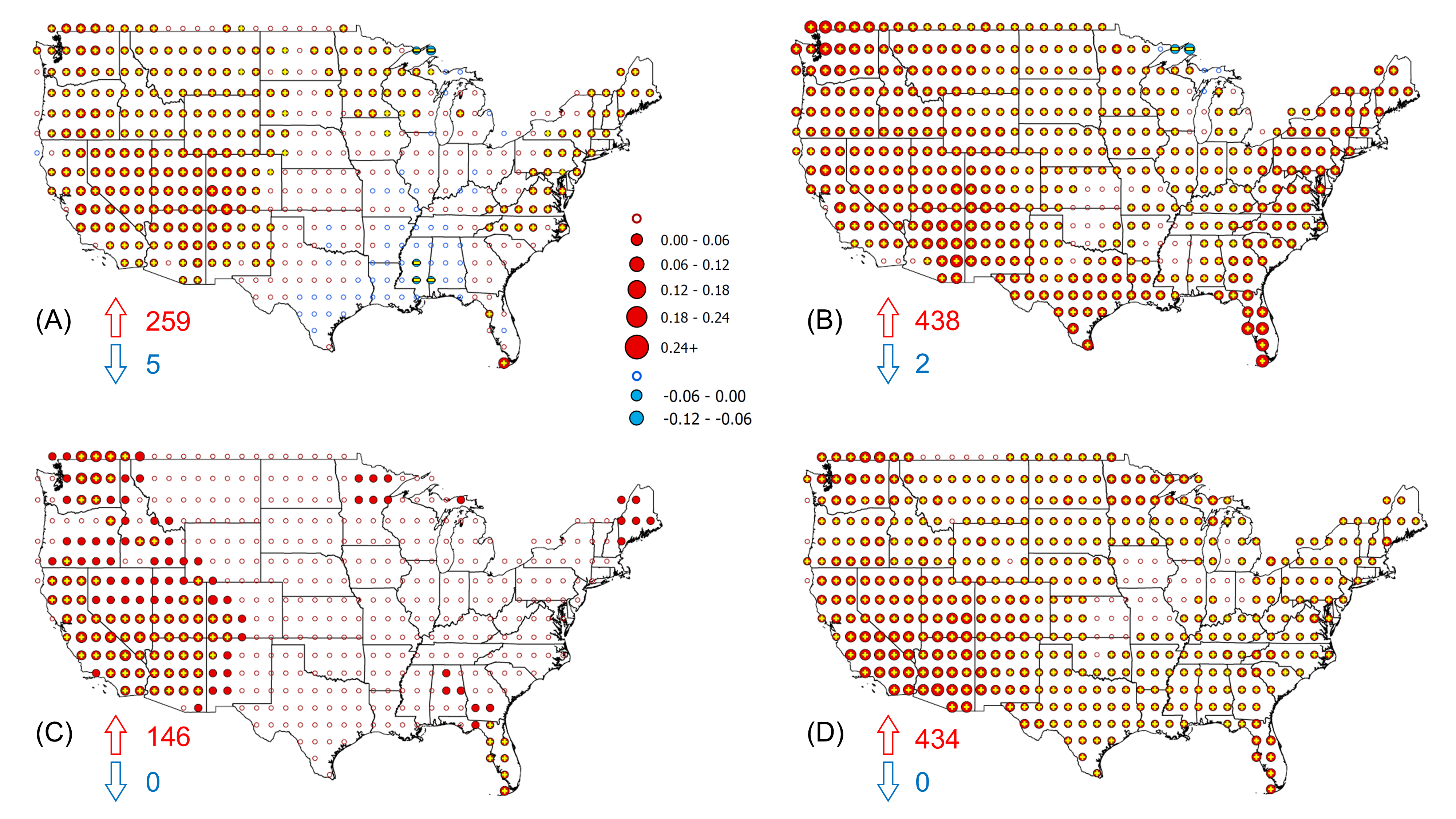

Supplement: S5 Fig — Base map from ESRI (VGIN, Esri, HERE, Garmin, FAO, NOAA, USGS, EPA, NPS; url: https://www.arcgis.com/home/item.html?id=979c6cc89af9449cbeb5342a439c6a76). (TIF) [file pone.0352866.s005.tif]

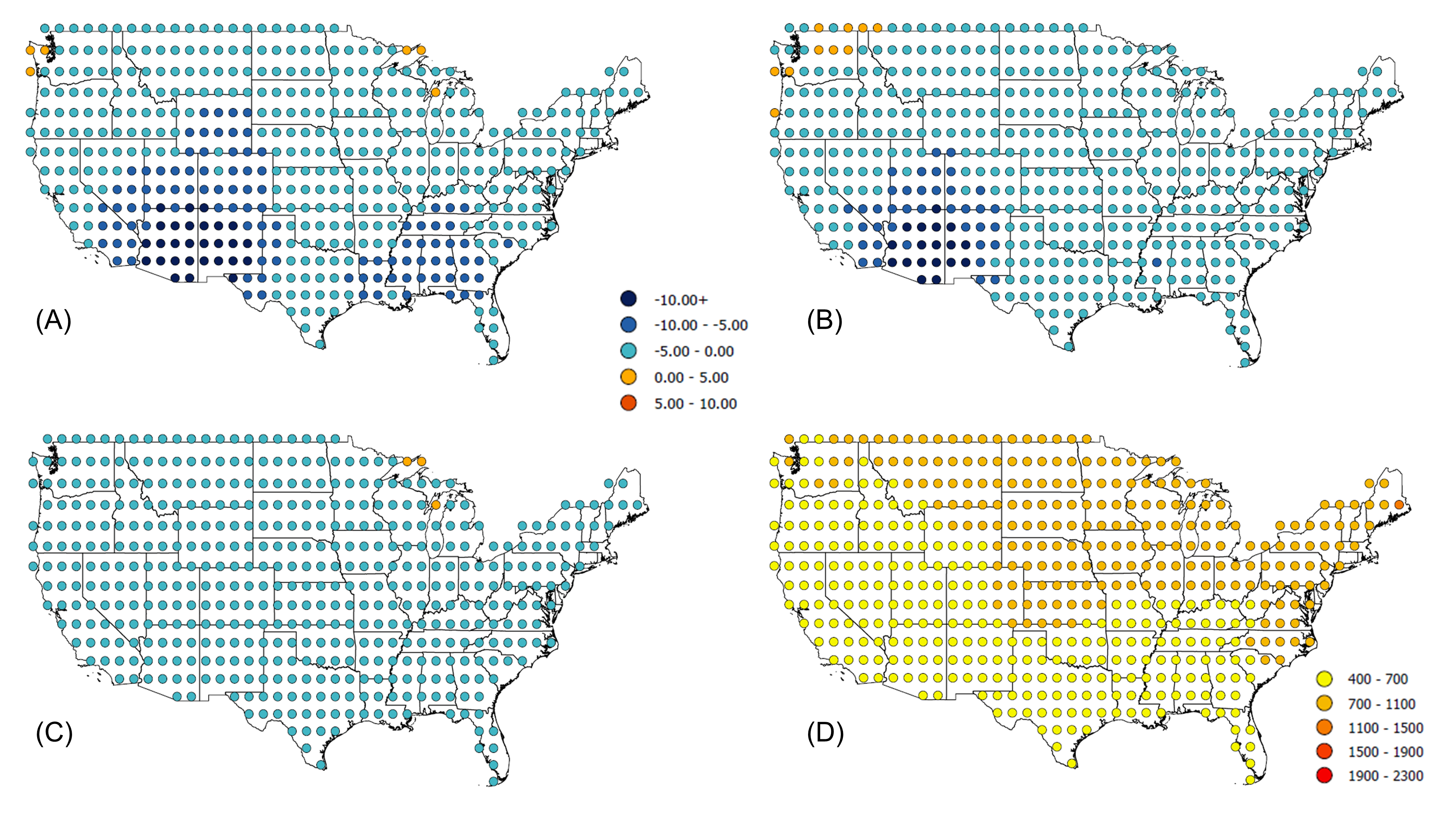

Supplement: S6 Fig — Base map from ESRI (VGIN, Esri, HERE, Garmin, FAO, NOAA, USGS, EPA, NPS; url: https://www.arcgis.com/home/item.html?id=979c6cc89af9449cbeb5342a439c6a76). (TIF) [file pone.0352866.s006.tif]

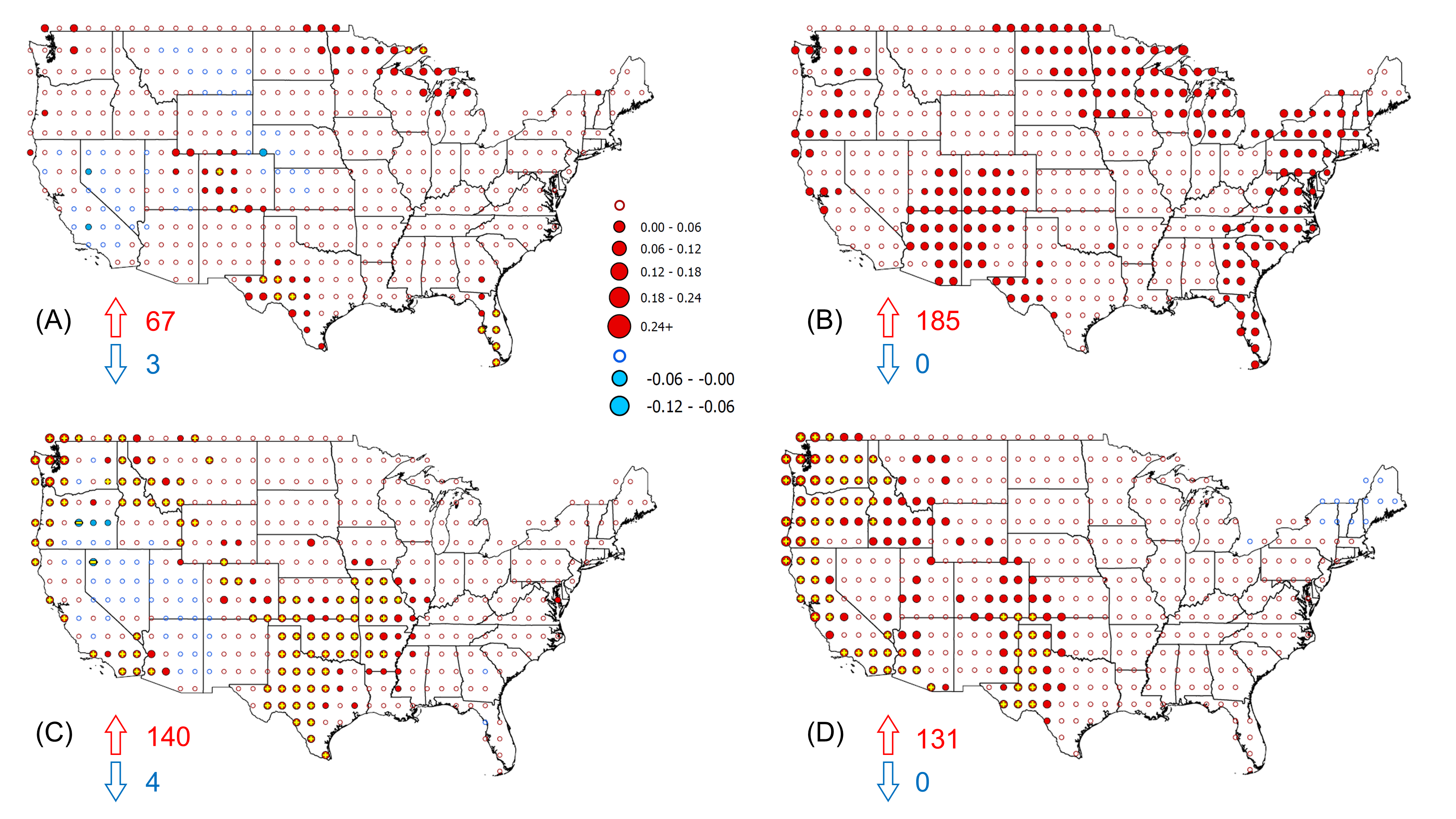

Supplement: S7 Fig — Base map from ESRI (VGIN, Esri, HERE, Garmin, FAO, NOAA, USGS, EPA, NPS; url: https://www.arcgis.com/home/item.html?id=979c6cc89af9449cbeb5342a439c6a76). (TIF) [file pone.0352866.s007.tif]

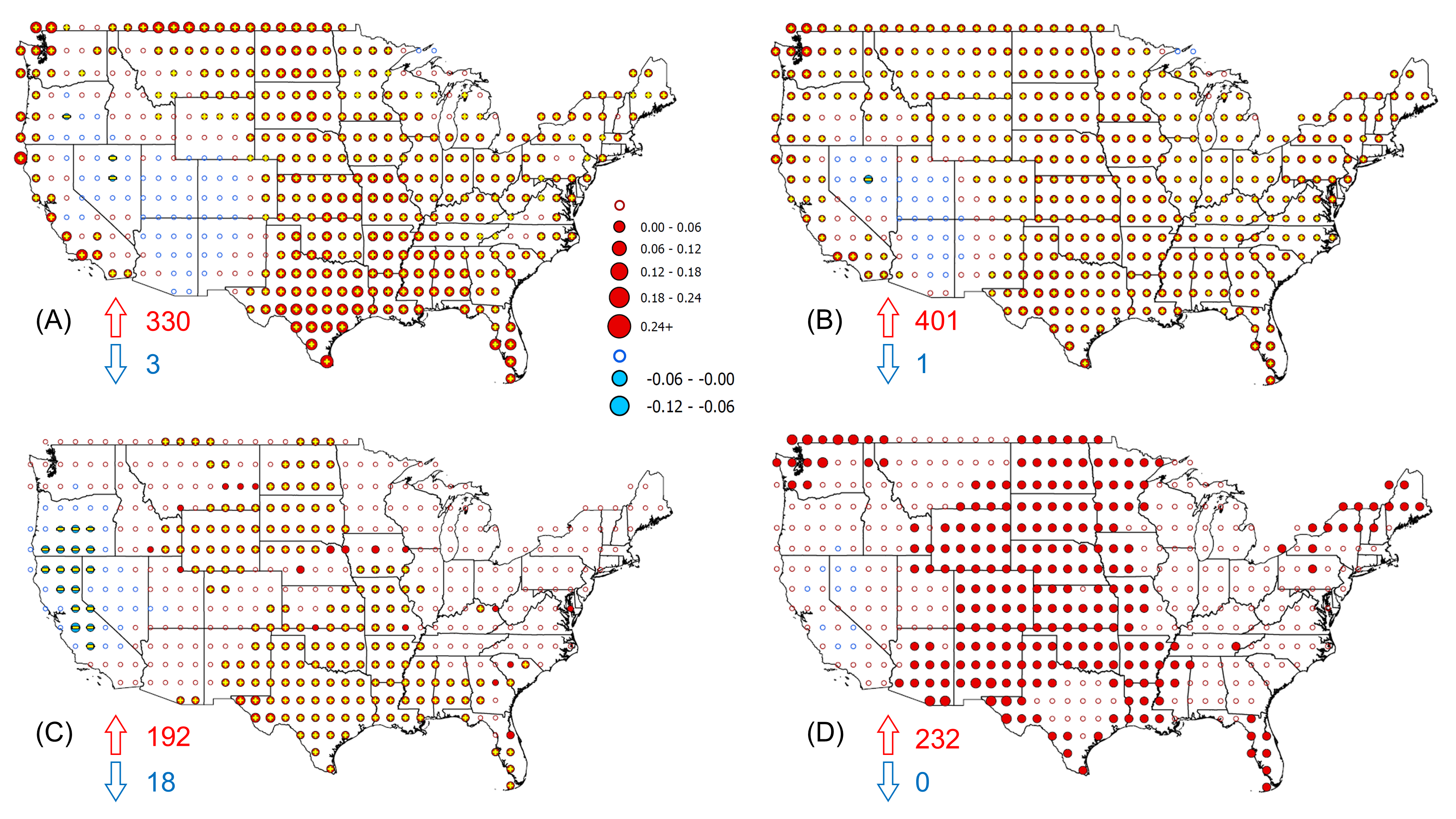

Supplement: S8 Fig — Base map from ESRI (VGIN, Esri, HERE, Garmin, FAO, NOAA, USGS, EPA, NPS; url: https://www.arcgis.com/home/item.html?id=979c6cc89af9449cbeb5342a439c6a76). (TIF) [file pone.0352866.s008.tif]

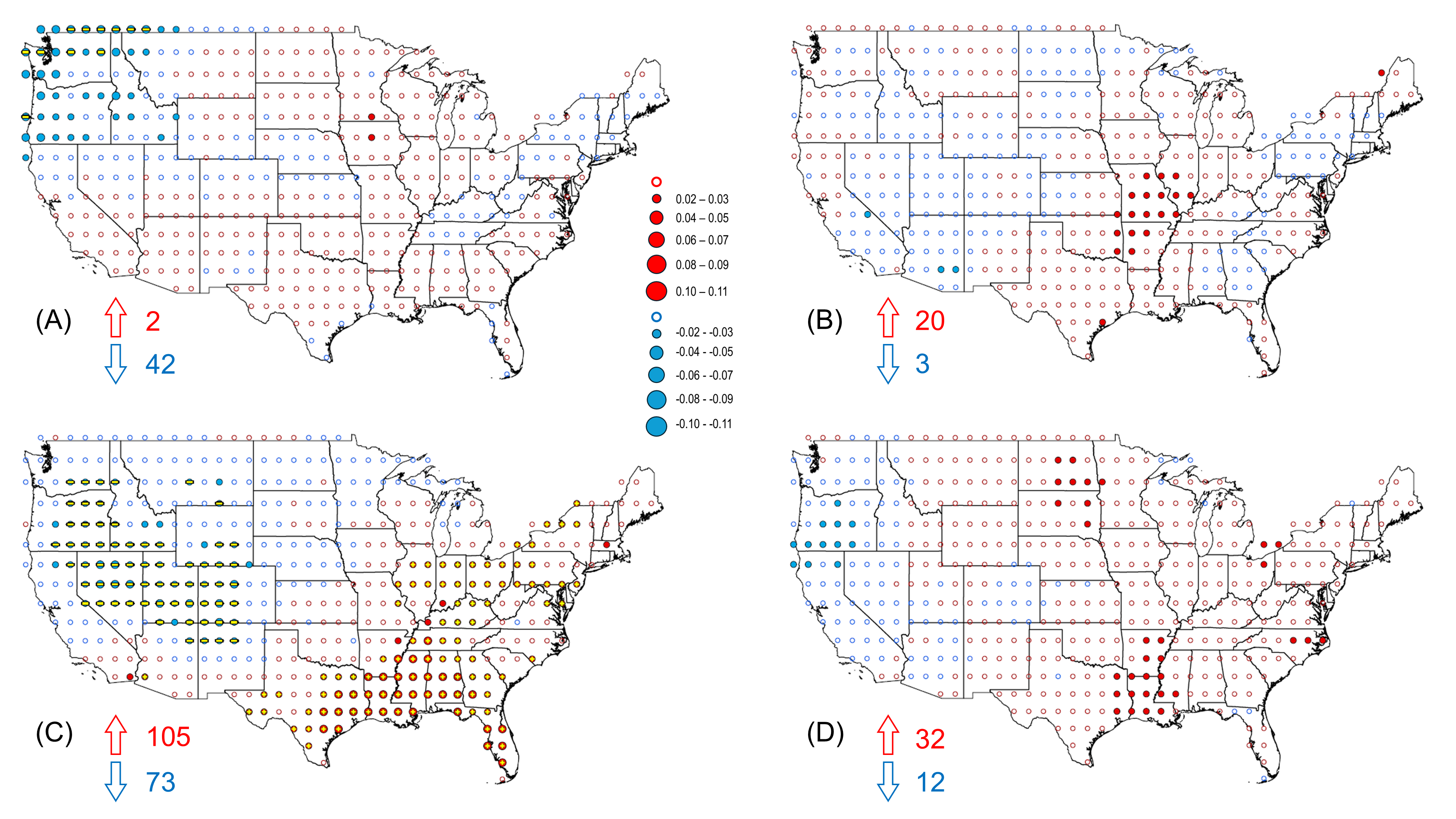

Supplement: S9 Fig — Plus and minus signs indicate those grid nodes that passed the FDR test that accounts for spatial autocorrelation. Base map from ESRI (VGIN, Esri, HERE, Garmin, FAO, NOAA, USGS, EPA, NPS; url: https://www.arcgis.com/home/item.html?id=979c6cc89af9449cbeb5342a439c6a76). (TIF) [file pone.0352866.s009.tif]
